# The Dental Pulp—Its Pathology and Treatment

**Published:** 1882-04

**Authors:** M. H. Chappelle

**Affiliations:** Professor Pathology and Therapeutics of Indiana Dental College


### THE DENTAL REGISTER.

VOL. XXXVI.] APRIL, 1882. [No. 4.

### THE DENTAL PULP—ITS PATHOLOGY AND TREATMENT.

BY M. H. CHAPPELLE, D.D.S.

PROFESSOR PATHOLOGY AND THERAPEUTICS OF INDIANA DENTAL COLLEGE.

[Read before the Mississippi Valley Dental Society March 6, 1882.]

(Published by request of the Association.)

*Mr. President, and Members of the Mississippi Valley Dental Association:*

GENTLEMEN:—In accepting an invitation from your committee to prepare an essay on the subject, "The Dental Pulp, its Pathology and Treatment," it could not be expected that a reflex summary of the combined wisdom of this paternal association of dentists should be given.

I shall endeavor to submit what I believe to be the true pathology and treatment of the dental pulp. If my efforts shall fall short of the task, I shall borrow consolation, and take refuge in that formal apology, "it will be an error of the *head*, and not of the heart."

It is a plain proposition in medical science, that perverted physiological function induces pathological conditions.

In the healthy functional exercise of any organ or tissue, the vital phenomena, as recognized in nutrition and waste, when normal, we have ease and are unconscious of the manifestation. But with the slightest disturbance, even the prick of a thorn through the cuticle, the first pathological symptom is experienced, that of pain, which calls immediately for relief, and if therapeutical surgery, in its simplest form, be not resorted to, that is, removal, the full primary train of pathological conditions will ensue, until suppuration and ulceration carries the thorn to the surface, or the parts die in the attempt.

A grain of sand imbedded in the conjunctiva, or chemical encroachment along the tubuli of the dentine, will provoke like results, and similar treatment is indicated.

The history of the dental follicle, or germ life, we shall omit, and take our study when the pulp is surrounded with its walls of dentine, having completed its histological development, and accepting the field of physiological function.

During anti-natal, as well as natal life, the structures that are being built up partake of and are subject to systemic tonicity, nourishment, or any dyscrasia, as formed in the protoplasm furnished.

The structure of the enamel, dentine and external soft parts contiguous thereto, indicate the pulp formation, its tonicity and susceptibilities.

Atrophied enamel, shriveled edges, with irregular enamel, leave their counter relations in the pulp.

If we judge from the variance in the different reports from old authors upon the cause of dental caries, we might be led to conclude that their investigations were erroneous, unsatisfactory and doubtful. While, with our present advantages in the way of improved microscopes, and knowledge in their use, with thoroughness in tabulating the sections made from various classes of teeth, and under different conditions, we find the types are as distinct in development as in external appearance, we here find why authors have varied in their conclusions.

The separate classes of reports have their peculiar abnormal morbid cachexia marked development, while others are perfect.

Mr. Bell advances the opinion that "caries is the result of inflammation in the dentine itself."

Mr. Fox maintains that "separation of the pulp membrane from the pulp cavity is the cause of caries."

Ficinus attributed dental caries to a "putrefaction produced by the minute infusorial animalcules, which live in the mouth and to which he has given the name of *denticola.*."

M. Klencke says that he "distinguishes *central caries* from common *peripheric* caries; the former commences in the cavity of the pulp, the latter in the external portions of the teeth." He subdivides this last class into three divisions.

"1st. A soft caries, caused by putrefaction.

"2d. A soft caries, due to the proliferation of a vegetable parasite called *Protococus dentalis.*

"3d. So called dry caries is caused by chemical action of acids upon the dental tissues."

Mr. J. Tomes holds that "acids are the principal cause of dental caries."

M. E. Magitot holds to the same in his works of 1868.

Mr. K. Bridgeman, about the same time, demonstrated that teeth were destroyed by electrolysis. He also says: "not only the destruction, but the formation of the teeth, are by the same law."

Leber and Rottenstein have labored assiduously to establish what they believe to be the true theory of caries, and rest equipoise between the *acid* and the *fungi*, or vegetable parasite theory.

With all these theories we find none more satisfactory than the selections hereinafter mentioned, and make a few observations as to the pathology of the pulps of the separate classes.

To formulate *rules* for *clinical* practice that might be found perfect in every particular, where such a variety of conditions and susceptibilities govern, would be impossible.

The most common cause of disease with the pulp arises from caries.

With the classification of teeth and caries—with a knowledge of constitutional tendencies before us, we can frame a reasonable prognosis of conditions.

We here submit a classification of teeth formulated by the late Dr. A. C. Castle, of New York.

1st. Large *dense yellow* teeth.

2d. *Dense* yellow-*white.*

3d. A — *Chalky*-white teeth.

B — *Chalky* dead-*yellow.*

4th. A — *Semi-transparent* yellowish *white.*

B — *Dead* chalk-*white.*

C — *Bluish-white pearly* teeth.

Also, with classification of caries, from Tomes, Watt, and others, with notes.

1st. Black = (*e.g.*) Indicating good normal development
(*Sulphuric Acid*) of tooth structures, with power to resist the
$H_2 S 0_4$ active elements of disintegration, and the secretions of the oral cavity normal.

2d. A — Dark-brown,
B — Shades of light-brown.
(*e.g.*) = A = Modification of the black decay.
B = The patient of apparent good constitution, but the oral secretions abnormal, with indications of anæmia, or mal-nutrition.
H C L.
(Hydrochloric acid)

3d. = Shades of yellow, with semi-cartilaginous residuum,
H C L. tubules of dentine remaining.
Abnormal constitutional tendencies, anæmia, with impaired nutrition in the dentine. Phthisis pulmonalis, scrofulosis, syphilis, and also with *senile* conditions.

4th. White decay.
H NO 3 The decomposition of nitrogenious substances,
(*Nitric Acid*) forming Nitric acid.—Caseous matter lodging between teeth and in the fissures of the crown, and lactic acid forming. Weak constitution, dyscrasia, or mark of intermixed race degeneration, with mal-nutrition, the powerful action of, formation of nascent acids.

Morbid alterations of the pulp exist in many cases, even where caries does not furnish the external existing cause.

Mal-nutrition and zymotic diseases during the period of development, or inherited cachexia, primarily produce or lead to atrophy, colloid or mucoid deposits, fatty infiltrations, or calcific granules, which may terminate in gangrene.

With patients where these conditions obtain we can observe external evidences; with the teeth in the way of opaque brown or yellow spots, shriveled or pitted appearing enamel, large crowns with deep incisor and canine fossa, high palate, or deep roofs, with mal-occlusion, indicating small defective tooth roots.

These morbid conditions are associated, and in these teeth we may look for *internal caries*, so called.

Therapeutical aid locally applied for the conservation of the pulp, is doubtful in these cases.

The third and fourth classes of caries, are associated with the third and fourth classes of teeth as a rule, and the pulps of these teeth suffer most from irritation, and are subject to all the phenomena leading to inflammation (*pulpitis*) resulting in various terminations.

1st. Hypertrophy, or osteo-dentinal cell deposits in the canaliculi between tubules.

2d. Calcific infiltration, in or at the mouth of the tubules.

3d. Resorption of dentine, (during gestation and lactation).

4th. Osteo-dentinal deposits with calcific inpactation, sometimes called secondary dentine, and pulp calcification in senile teeth.

5th. Granules in the parenchyma of the pulp.

6th. Colloid and Mucoid deposits, and net-like atrophy.

7th. Gangrene, soft, or dry.

8th. Suppuration and putrescence.

In most cases, the activity of the pulp organs, with proper tonicity of the general system, with the irritation controlled, will, with young persons, decrease the size of the pulp, and solidify its succulent condition, and develop the structure from indolence and abnormal susceptibilities to that of mature function.

Therefore, the utmost necessity of allaying nerve irritation, and relieving the determination of blood in the parts is required.

In plethoric patients, antiphlogistic treatment — arterial and nerve sedatives may be resorted to with great benefit.

In dyspeptic or anæmic patients, tonics with nerve stimulants should be administered.

Carious dentine, which, if being left, would prove a basis for active chemical or parasitical phenomena, should be removed, excepting when in the *third class of carious* cavities, where the fibrils are thought to be alive.

Then antiseptic (not escharotic) treatment, will remove the irritating and disintegrating tendency, and the cavity can be filled with a non-irritating material, and be successful.

There are comparatively few cases of exposed pulps without previous irritation, and great pain where the pulp membrane is bare, in comparison with the non-exposed cases.

Exposures in this sense most frequently terminate in morbid growths. Hernia and epulides most frequently result in the death and putrescence of the pulp.

In many cases of hernia the application of iodine and aconite will stimulate and reduce the hernia, and if thought free from colloid or mucoid deposits or granules, a dressing of tannate of glycerine and oxide of zinc, with a temporary filling, will in ten days find the pulp reduced to its normal condition, when the dressing of dry phosphate of zinc will prove satisfactory.

Abscess of the pulp will, when the cavity is filled imperfectly, result in resorption of the disintegrated wall, or any substance in contact with the abraded pulp membrane or terminal fibrils, metamorphosing into a lamella of granules, or until a small orifice (*tap hole*) appears in the carious cavity.

And when the vital force of the patient is normal the pulp will resume healthy action with cicatrical membrane exposed, and, with proper *dry dressing* of phosphate of zinc and filling the cavity properly, the pulp will maintain healthy function, and calcific granules *will become impacted into the dressing*, forming a lamella compatible with the pulp structure.

Morbid growths are most likely to be found in the third and fourth class of teeth, and the growth is determined by the cachexia of the patient, when malignant, and the nature of the irritant.

The treatment for the conservation of the pulp tissue, as practiced by many, would be to apply oxichloride of zinc or carbolic acid, full strength, and then fill the cavity. Saying to the patient, " we will see what it will do, if it hurts you too bad come back." It is often the case, some other dentist attends to that tooth, and has it on exhibition as a trophy.

The pulp being subject to many pathological conditions, as heretofore indicated, and subject to constitutional tendencies, as exciting, and the carious exposure the predisposing cause.

While in other cases, and most frequently, the carious exposure would be exciting, and the constitutional dyscrasia the predisposing cause, and the treatment vary accordingly.

Hence, we find that for the better treatment of the pulp, and consequently the tooth, the dentist should be well versed in general pathology and the application of therapeutical remedies, thereby giving the greatest relief to suffering humanity.

It is to be regretted that dental pathology and therapeutics are not taught to any satisfactory extent in the medical colleges of this country.

And if it should be thought to be presumption on the part of the dentist to advocate general pathology and therapeutics in connection with dental practice, we take refuge in the thought, that it is all for the sake of humanity.

When we are fully advised as to the true condition of the pulp, and knowing that systemic tonicity controls or holds to any extent the destiny of this highly vascular organ, and failing to resort to general medication in connection with topical applications, we fall short of our professional usefulness.

The practice of *applying escharotics indiscriminately* to all classes of pulp indications, whether acid irritation, atrophy, hypertrophy, congestion, inflammation, calcification, suppuration, or putrescence, is undoubtably questionable.

We know that carbolic acid is antiseptic, disinfectant, tonic and alterative, owing to the formula, while it is a powerful escharotic, destroying cell and infusorial life in the dentinal tissues, or other parts, and the whole pulp structure, if confined in contact.

Hence, when judiciously used, it is invaluable. Otherwise, it is destructive.

In *acid irritation*, instead of *liquid* treatment, use hot air and dry dressing of oxyde or phosphate of zinc, or oxide of tin dry white lead, or sulphate of lime, and if the teeth belong to the third or fourth class, and third or fourth class of caries, a temporary filling of gutta-percha, or oxyphosphate of zinc is absolutely necessary.

From six to twelve months, osteo-dentine deposits, or calcific infiltration will thoroughly protect the pulp, and a gold or other metal filling can be substituted, if desired.

Pulpitis or local hyperemia may be determined by the length of time the first pain symptoms were manifest, and the continuance decides as to simple *fibril irritation.* Sharp pain by tapping the tooth if primarily acute, hot application to the tooth will relieve the pain, if in a progressive stage it will increase the pain. Cold applications giving momentary relief; when reaction takes place pain will increase, while in some cases cold application gives complete relief.

We will now consider the phenomena leading to inflammation.

First with irritation, determination of blood in the pulp, with stasis in the capillaries, or parenchyma (congestion). Now, when the arterial action forces the fluids through to the venous sinuses, and the increasing irritation causing an exalted flow of blood, and the blood corpuscles sticking to the walls of the capillary canals and liquor sanguinis, or lymph, passing through these delicate sheaths by osmotic action, we then have effusion in the connecting tissue, and hyperæmia is now at high tide, and if not arrested by systemic and local medication, effusive granules and then exudate corpuscles will develop, and finally organize into pus corpuscles, when septicæmia or pyæmia continues until suppuration terminates the pulp, and the abscess discharges through the apical foramen, and periodental abscess follows until ulceration discharges the pus through the alveolar walls.

I am aware that with many practitioners the conservation of the pulp, after being once diseased, and the retention of pulpless teeth by proper treatment, is by them considered of doubtful practicability. I have this to say, as upon the question of Christian character and life, to those who condemn or doubt, have they squared their lives by the teaching of the scripture? Have they conformed their practice to the highest conceptions of physiological, pathological, and therapeutical laws governing the case?

It is reasonable to suppose that the blood that flows into the pulp is supplied with protoplasm, to be converted into bone, corpuscles or cells, and the dentinal cells or odontoblasts appropriate by action similar to the lactæls, or secrete like a gland, simple and compound, and properly distributed throughout the tubuli, and transverse tubular structure.

If the nerve fibrils be irritated to such an extent that deposits of osteo-dentine takes place, the irritation will only be a tonic, and of good results.

While if the tubules be short and the caries in near approach to the pulp, the irritation will be violent, and result in all the phenomena leading to inflammation spoken of, provided calcification does not cut off the line of irritation, as found in senile teeth, which includes chemical and mechanical abrasion.

We are aware that meningitis, peritonitis, pleuritis, and periostitis are considered difficult conditions to remedy.

But the responsibility of neglect, and consequences so disastrous, that rational consideration and relief is imperative, and the treatment of *pulpitis* may be equally so regarded.

The teeth should be kept dry by the use of rubber dam, or by napkins, when treatments are made to the pulps, or when fillings are made. And all treatments should be securely sealed with plastics, either gutta-percha or oxyphosphate of zinc.

And the operations should be as neat and delicate as the highest conception of the operator will admit.

*Pulp treatments* should not be confounded with the application of carbolic acid, sulphuric acid, permangenate of potash, chloride of zinc and iodine, with putrescent pulps, or pulp canal treatments, when periodontal abscesses discharge through the canal.

Pulp irritation, caused by fibril exposure, pain of short duration, rather intermittent, can usually be successfully remedied by dry dressing and appropriate filling, as heretofore explained.

For the relief of acute pulpitis apply a paste over the pulp, in the carious cavity of the following.

(No 1.) ℞ Atropiæ sulphas gr j

Plumbi acetas gr ij

Tinct opii q. s.

Thoroughly seal the cavity.

For internal use when aggravated by cold, or miasma prescribe.

(No 2.) ℞ Quinia sulphas } $\bar{a}\,\bar{a}$ *g* XV

Dovers powders }

Make into five powders; take one every three hours.

A satisfactory indication with the constitutional tendencies is to observe if there be any abrasions of the cuticle or incised wounds, and that they heal by first intention instead of by inflammatory granulations.

If the patient be plethoric, active cathartics should be given until free discharges are effected, and at the same time give the following:

℞ Tinc aconiti rad gtt XX
Acid sulphuric gtt ij
Aqua ℥ ij

Misce; dose, teaspoonful every hour.

A topical application over the tooth, on a pledget of cotton of the following:

℞ Fl. ex. belladonæ

Tinct aconiti rad

Tinct opii

Tinct camphora } a a ʒj

Misce:

When the pain subsides omit the medication, or when the constitutional effects of the drugs are manifested. Time will not permit me to extend my remarks upon this subject. Much remains unsaid.

With these suggestions, and a fair trial by those who do not resort to general medication, dry dressings, with less escharotics, we feel assured that their success in saving tooth pulps will greatly increase.

We have said nothing about the use of *arsenious acid* in the destruction of pulps, *and wish less could be said.*

We cannot always control our patients, some of them want ease immediately, they, with the same eagerness want money, learning, reputation, and are off without warning, to the other world on the same reckless time. They are also indifferent as to their teeth, body or soul, and care little whether tartar, bacteria, last week's bill of fare, pulpless teeth, or cheap store teeth, occupy the oral cavity.

With such patients arsenic is indicated, and even cold steel will meet the requirement.

If I have said anything that may prove a benefit to any of my hearers, I have been paid for my efforts, and if I have fallen short of the task assigned me, or have offended, I can say in the language of my friend Dr. Jennings, "If I have offended any one, I am perfectly willing to be forgiven."

I thank you for your kind and patient attention.

